# Impact of Race and Socioeconomic Status on Outcomes in Patients Hospitalized with COVID-19

**DOI:** 10.1007/s11606-020-06527-1

**Published:** 2021-01-27

**Authors:** Daniel Quan, Lucía Luna Wong, Anita Shallal, Raghav Madan, Abel Hamdan, Heaveen Ahdi, Amir Daneshvar, Manasi Mahajan, Mohamed Nasereldin, Meredith Van Harn, Ijeoma Nnodim Opara, Marcus Zervos

**Affiliations:** 1 Wayne State University School of Medicine, Detroit, MI USA; 2 Department of Infectious Disease, Henry Ford Hospital, Detroit, MI USA; 3 Department of Public Health Sciences, Henry Ford Hospital, Detroit, MI USA; 4 Department of Internal Medicine, Internal Medicine-Pediatrics Section, Wayne State University School of Medicine, Detroit, MI USA; 5 Global Affairs Professor of Medicine, Assistant Dean Wayne State University School of Medicine, MI Detroit, USA; 6 Infectious Diseases, Division Head Henry Ford Health System, MI Detroit, USA

**Keywords:** COVID-19, disparities, disadvantage, socioeconomic status, race

## Abstract

**Background:**

The impact of race and socioeconomic status on clinical outcomes has not been quantified in patients hospitalized with coronavirus disease 2019 (COVID-19).

**Objective:**

To evaluate the association between patient sociodemographics and neighborhood disadvantage with frequencies of death, invasive mechanical ventilation (IMV), and intensive care unit (ICU) admission in patients hospitalized with COVID-19.

**Design:**

Retrospective cohort study.

**Setting:**

Four hospitals in an integrated health system serving southeast Michigan.

**Participants:**

Adult patients admitted to the hospital with a COVID-19 diagnosis confirmed by polymerase chain reaction.

**Main Measures:**

Patient sociodemographics, comorbidities, and clinical outcomes were collected. Neighborhood socioeconomic variables were obtained at the census tract level from the 2018 American Community Survey. Relationships between neighborhood median income and clinical outcomes were evaluated using multivariate logistic regression models, controlling for patient age, sex, race, Charlson Comorbidity Index, obesity, smoking status, and living environment.

**Key Results:**

Black patients lived in significantly poorer neighborhoods than White patients (median income: $34,758 (24,531–56,095) vs. $63,317 (49,850–85,776), *p* < 0.001) and were more likely to have Medicaid insurance (19.4% vs. 11.2%, *p* < 0.001). Patients from neighborhoods with lower median income were significantly more likely to require IMV (lowest quartile: 25.4%, highest quartile: 16.0%, *p* < 0.001) and ICU admission (35.2%, 19.9%, *p* < 0.001). After adjusting for age, sex, race, and comorbidities, higher neighborhood income ($10,000 increase) remained a significant negative predictor for IMV (OR: 0.95 (95% CI 0.91, 0.99), *p* = 0.02) and ICU admission (OR: 0.92 (95% CI 0.89, 0.96), *p* < 0.001).

**Conclusions:**

Neighborhood disadvantage, which is closely associated with race, is a predictor of poor clinical outcomes in COVID-19. Measures of neighborhood disadvantage should be used to inform policies that aim to reduce COVID-19 disparities in the Black community.

**Supplementary Information:**

The online version contains supplementary material available at 10.1007/s11606-020-06527-1.

## INTRODUCTION

As of October 23, 2020, there are more than 8.3 million confirmed cases and 221,000 deaths from coronavirus disease 2019 (COVID-19) in the USA.^1^ Non-White Americans, especially Black Americans, have been disproportionately affected by the pandemic. In the state of Michigan, Black Americans represent 37% of COVID-19 cases and 42% of deaths, despite making up 14% of Michigan’s population.^2–4^ These discrepancies have been largely attributed to social and health disparities rendering Black Americans particularly vulnerable to this novel coronavirus. This explanation is supported by reports that most patients who have been hospitalized and died from COVID-19 have medical comorbidities (e.g., hypertension, diabetes, chronic obstructive pulmonary disease (COPD), heart disease, obesity) that are disproportionately prevalent in the Black community.^4–8^

The effects of medical comorbidities, age, and sex on prognosis in COVID-19 have been widely examined and replicated in previous studies.^5,7–10^ However, systematic examination of race and socioeconomic underpinnings of the COVID-19 pandemic has been lacking. One study showed an increase in the likelihood of hospital admission for COVID-19 in Black individuals and those who lived in low-income areas.^7^ Socioeconomic disadvantage is multifaceted, encompassing economic, educational, social, housing, and healthcare disparities. When combined with patient-level risk factors, neighborhood socioeconomic variables have been used to increase the accuracy of predictive models, including risk of preterm birth,^11^ depression,^12^ and cardiovascular disease mortality.^13^

Research into how social determinants can shape COVID-19 outcomes is needed. Our study seeks to fill this gap by examining race and patient-level socioeconomic variables, including social support, living environment, and employment status. The objective was to determine whether race and socioeconomic status were associated with clinical outcomes in patients hospitalized with COVID-19. We analyzed the association between patient sociodemographics and census tract socioeconomic variables and frequencies of death, invasive mechanical ventilation (IMV), and intensive care unit (ICU) admission in hospitalized patients. Our aim is to contribute new data that might help inform policies to reduce disparities in the health system.

## METHODS

### Study Design and Participants

This is a retrospective cohort study approved by the Henry Ford Health System Institutional Review Board (IRB #13843). The Henry Ford Health System is a large, integrated healthcare system serving southeast Michigan. Eligible patients were adult patients admitted to four large hospitals within the Henry Ford Health System from March 12, 2020, to April 24, 2020, inclusive of these dates, and had a positive SARS-CoV-2 test by qualitative polymerase chain reaction. This time period encompasses the rise and peak of the first surge of the pandemic in southeast Michigan. For patients with multiple admissions within the study period, data from the longest admission was presented due to a higher probability of experiencing the clinical outcomes of interest. Patients were followed until May 27, 2020, inclusive. Follow-up encounters consisted of phone calls, video, and in-person visits which verified patients’ survival status.

### Data Collection

Clinical data were extracted from the Henry Ford electronic medical record through patient chart review. Our data extraction included the following: patient demographics (age, sex, race, ethnicity, English language proficiency, insurance status, and living situation), employment status for patients ≤ 65 years, smoking status, substance use, body mass index (BMI), comorbidities, and clinical outcomes. Essential industries included healthcare, agriculture, food service, first responders, transportation, infrastructure, and critical manufacturing.^14^ Medical comorbidities and the Charlson Comorbidity Index (CCI) were obtained by examining the medical history, initial history, and outside care records.

Census tracts from patient addresses were used to obtain socioeconomic data from the 2018 American Community Survey and the Food Access Research Atlas,^15,16^ data which has been validated and used in health disparities research.^17–19^ The poverty rate obtained from the Food Access Research Atlas is defined as the share of the tract population with income at or below federal poverty thresholds by family size. Census tract median income was used as a numerical surrogate for neighborhood socioeconomic status because of its ease of interpretation and significant correlation with other neighborhood socioeconomic variables.

Presenting symptoms, vital signs, initial laboratory values, and hospital course data were also collected and provided in Appendix Tables 2–5.

### Outcomes

The primary outcomes were death, invasive mechanical ventilation, and ICU admission anytime during hospitalization.

### Statistical Analysis

Continuous variables were described as mean (SD) or median (Q1–Q3). Categorical variables were described with counts and percentages. Univariate comparisons between groups were carried out using independent two-group *t* tests for normally distributed continuous variables and using Wilcoxon rank-sum tests for non-normally distributed continuous variables. Categorical variables were compared using chi-square or Fisher’s exact test based on expected cell count. Multivariable logistic regression models were used to identify possible independent predictors of death, ventilator use, and ICU admission with results presented as adjusted odds ratios and 95% confidence intervals. Increased age, male sex, Black race, increased comorbidity burden, obesity, and smoking were incorporated into the multivariable model because they have been associated with death and severe disease in previous studies.^7–10^ When post hoc pairwise testing was performed, the type I error rate was controlled using a Benjamini-Hochberg correction and adjusted *p* values were reported. All analyses were performed using SAS 9.4 (SAS Institute Inc, Cary, NC, USA).

## RESULTS

### Patient Demographics

Of the 2038 patients hospitalized, 1209 (59.3%) were Black and 694 (34.1%) were White. The mean age of the hospitalized patients was 64 years, and approximately half were female (Table 1). Black patients comprised a larger proportion of each hospital’s patients than the surrounding city’s Black population. The proportion of Black patients was 90.2% in Henry Ford Hospital, 35.5% in Henry Ford Macomb, 53.0% in Henry Ford West Bloomfield, and 39.0% in Henry Ford Wyandotte. Conversely, census data shows the Black population comprises 77% of the population in Detroit, 18% in Macomb, 13% in West Bloomfield, and 1% in Wyandotte.^20^

**Table 1:** Socioeconomic and Environmental Characteristics of Alive and Dead Patients Hospitalized with COVID-19

Characteristic	All (*n* = 2038)	Alive (*n* = 1587)	Dead (*n* = 442)
Age, mean (SD), years	63.96 (16.23)	60.9 (15.6)	75.1 (13.6)
Female, no. (%)	1027 (50.4)	824 (51.95)	201 (45.48)
Length of stay, days (Q1–Q3)	6 (3–12)	6 (3–10)	9 (4–15)
Readmission, no. (%)	226 (11.1%)	172 (10.84)	54 (12.22)
Race, no. (%)			
White	694 (34.1)	491 (30.94)	201 (45.48)
Black	1209 (59.3)	984 (62)	218 (49.32)
Asian/Pacific Islander	39 (1.9)	32 (2.02)	7 (1.58)
Native American	2 (0.1)	1 (0.06)	1 (0.23)
Other/missing	94 (4.6)	79 (4.98)	15 (3.39)
Living situation, no. (%)			
With family	1210 (66.2)	969 (69.86)	234 (54.29)
Alone	267 (14.6)	217 (15.65)	49 (11.37)
Group facility	350 (19.2)	201 (14.49)	148 (34.34)
Insurance status, no. (%)			
Commercial insurance	494 (26.9)	461 (32.24)	32 (8.04)
Medicare	958 (52.2)	629 (43.99)	324 (81.41)
Medicaid	304 (16.6)	268 (18.74)	35 (8.79)
Self-pay	4 (0.2)	4 (0.28)	0 (0)
Other	21 (1.1)	19 (1.33)	2 (0.5)
No insurance	54 (2.9)	49 (3.43)	5 (1.26)
Medical conditions, no. (%)			
BMI, mean (SD), kg/m^2^	31.75 (8.66)	32.41 (8.53)	29.3 (8.61)
Charlson Comorbidity Index, mean (SD)	2.22 (2.18)	1.84 (1.91)	3.57 (2.54)
Hypertension	1494 (73.3)	1121 (70.64)	364 (82.54)
Psychiatric condition	520 (25.5)	384 (24.2)	134 (30.39)
Coronary artery disease	308 (15.1)	197 (12.41)	108 (24.49)
Congestive heart failure	276 (13.5)	162 (10.21)	111 (25.17)
Cerebrovascular disease	271 (13.3)	163 (10.27)	107 (24.26)
COPD	294 (14.4)	176 (11.09)	115 (26.08)
Diabetes	652 (32.0)	490 (30.88)	157 (35.6)
Diabetes with complications	132 (6.5)	90 (5.67)	40 (9.07)
Chronic kidney disease	357 (17.5)	216 (13.61)	136 (30.84)
Malignancy	136 (6.7)	88 (5.55)	48 (10.88)
Metastatic cancer	26 (1.3)	10 (0.63)	16 (3.63)
Tobacco use	810 (43.1)	589 (39.96)	218 (54.64)
Substance use	186 (10.3)	146 (10.3)	39 (10)
Neighborhood characteristics, % (SD)			
Bachelor’s degree or higher	18 (13)	18 (13)	18 (13)
Less than high school	9 (6)	9 (6)	9 (5)
Median income (Q1–Q3)	47,645 (29,541–68,924)	47,363 (29,721–70,104)	49,850 (29,721–148,875)
Renters	37 (22)	37 (22)	37 (21)
Unemployment	5 (3)	5 (3)	5 (4)
Poverty rate	24 (18)	24 (18)	23 (17)
SNAP recipients	24 (17)	24 (18)	23 (17)
No vehicle access	14 (13)	14 (14)	14 (12)

*Q1–Q3*, first quartile–third quartile; *SD*, standard deviation; *BMI*, body mass index; *COPD*, chronic obstructive pulmonary disease; *SNAP*, Supplemental Nutrition Assistance Program

### Patient Outcomes

At follow-up, 1587 patients were discharged alive, 442 had died, and 9 were still admitted. A total of 405 patients died in the hospital (91.6%), of which 182 were White and 202 were Black. The median follow-up time for patients discharged alive was 27 days (3–37) and median time to death for patients who died outside the hospital was 12 days (6–23). The median duration of hospitalization for the nine patients still admitted was 46 days (46–49). The case fatality rate (CFR) for all patients was 21.7% and White patients had a higher CFR than Black patients (29.1% vs. 18.1%, *p* < 0.001) (Table 2). Conversely, Black patients were more likely than White patients to receive mechanical ventilation (23.6% vs. 19.7%, *p* = 0.053) and require ICU level care (31.4% vs. 25.5%, *p* = 0.003). Of the 455 patients who received mechanical ventilation, 215 (47.2%) were discharged alive, 231 (50.8%) died, and 9 were still admitted.

**Table 2:** Socioeconomic and Environmental Characteristics of Black and White Patients

Characteristic	White (*n* = 694)	Black (*n* = 1209)
Age, mean (SD), years	68.6 (16.7)	61.7 (15.5)
Female, no. (%)	341 (49.14)	635 (52.57)
Clinical outcomes, no. (%)		
Death	201 (29.05)	218 (18.14)
Mechanical ventilation	137 (19.74)	285 (23.57)
ICU admission	174 (25.07)	380 (31.43)
Length of stay, days (Q1–Q3)	6 (4–12)	6 (3–12)
Readmission, no. (%)	107 (15.42)	114 (9.43)
Living situation, no. (%)		
With family	381 (59.25)	744 (69.6)
Alone	65 (10.11)	184 (17.21)
In a facility	197 (30.64)	141 (13.19)
Insurance status, no. (%)		
Commercial insurance	151 (23.74)	305 (27.9)
Medicare	392 (61.64)	533 (48.76)
Medicaid	71 (11.16)	212 (19.4)
Self-pay	1 (0.16)	2 (0.18)
Other	7 (1.1)	12 (1.1)
No insurance	14 (2.2)	29 (2.65)
Medical conditions, no. (%)		
BMI, mean (SD), kg/m^2^	29.9 (7.8)	33.0 (8.9)
Charlson Comorbidity Index, mean (SD)	2.35 (2.18)	2.22 (2.18)
Hypertension	474 (68.4)	932 (77.09)
Psychiatric condition	232 (33.48)	268 (22.17)
Coronary artery disease	151 (21.79)	146 (12.08)
Congestive heart failure	104 (15.01)	162 (13.4)
Cerebrovascular disease	101 (14.57)	158 (13.07)
COPD	119 (17.17)	165 (13.65)
Diabetes	185 (26.7)	413 (34.16)
Diabetes with complications	37 (5.34)	92 (7.61)
Chronic kidney disease	112 (16.16)	235 (19.44)
Malignancy	52 (7.5)	73 (6.04)
Metastatic cancer	10 (1.44)	14 (1.16)
Tobacco use	322 (49.92)	463 (41.34)
Substance use	47 (7.62)	131 (12.01)
Neighborhood characteristics, % (SD)		
Bachelors or higher	22 (13)	15 (13)
Less than high school	7 (5)	10 (6)
Median income (Q1–Q3)	63,317 (49,850–85,776)	34,758 (24,531–56,095)
Renters	26 (19)	44 (21)
Unemployment	3 (2)	6 (3)
Poverty rate	13 (11)	30 (18)
SNAP recipients	13 (12)	30 (17)
No vehicle access	7 (8)	19 (14)

*Q1–Q3*, first quartile–third quartile; *SD*, standard deviation; *ICU*, intensive care unit; *BMI*, body mass index; *COPD*, chronic obstructive pulmonary disease; *SNAP*, Supplemental Nutrition Assistance Program

Patients who died were significantly older than those discharged alive (75.1 ± 13.6 years vs. 60.9 ± 15.6 years, *p* < 0.001) and had more comorbidities (CCI: 3.6 ± 2.5 vs. 1.8 ± 1.9, *p* < 0.001) (Table 1). They had higher rates of hypertension, psychiatric conditions, cardiovascular disease, COPD, chronic kidney disease (CKD), and cancer. White patients were significantly older than Black patients (68.6 ± 16.7 years vs. 61.7 ± 15.5 years, *p* < 0.001), although the CCI was similar among the two races (2.4 ± 2.2 vs. 2.2 ± 2.2, *p* = 0.21) (Table 4). White patients were more likely to have a history of coronary artery disease and psychiatric conditions. Black patients were more likely to have a history of hypertension, diabetes, and obesity. Patients who died had lower BMI, but those who received IMV and ICU care had higher BMI (Tables 1, 2, and 3). Presenting symptoms, vital signs, initial laboratory values, and hospital course data are provided in the Appendix. Patients with commercial insurance had lower rates of death, IMV, and ICU admission than those with Medicaid insurance. Those who lived in group facilities had a higher risk of death, but lower frequencies of IMV and ICU admission. A larger proportion of White patients lived in group facilities than Black patients (30.6% vs. 13.2%, *p* < 0.001) (Table 4).

**Table 3:** Socioeconomic and Environmental Characteristics of Patients Requiring Mechanical Ventilation

Characteristic	No IMV (*n* = 1596)	IMV (*n* = 442)
Age, mean (SD), years	63.5 (16.8)	65.5 (14.0)
Female, no. (%)	836 (52.41)	191 (43.21)
Length of stay, days (Q1–Q3)	6 (3–8)	19 (10–26)
Readmission, no. (%)	185 (11.59)	41 (9.28)
Race, no. (%)		
White	557 (34.9)	137 (31)
Black	924 (57.89)	285 (64.48)
Asian/Pacific Islander	29 (1.82)	10 (2.26)
Native American	2 (0.13)	0 (0)
Other/missing	84 (5.26)	10 (2.26)
Living situation, no. (%)		
With family	902 (64.75)	308 (70.97)
Alone	207 (14.86)	60 (13.82)
Group facility	284 (20.39)	66 (15.21)
Insurance status, no. (%)		
Commercial insurance	417 (29.2)	77 (18.92)
Medicare	709 (49.65)	249 (61.18)
Medicaid	238 (16.67)	66 (16.22)
Self-pay	4 (0.28)	0 (0)
Other	19 (1.33)	2 (0.49)
No insurance	41 (2.87)	13 (3.19)
Medical conditions, no. (%)		
BMI, mean (SD), kg/m^2^	31.3 (8.3)	33.3 (9.8)
Charlson Comorbidity Index, mean (SD)	2.06 (2.10)	2.81 (2.36)
Hypertension	1131 (70.91)	363 (82.13)
Psychiatric condition	402 (25.2)	118 (26.7)
Coronary artery disease	218 (13.67)	90 (20.36)
Congestive heart failure	193 (12.1)	83 (18.78)
Cerebrovascular disease	207 (12.98)	64 (14.48)
COPD	199 (12.48)	95 (21.49)
Diabetes	471 (29.53)	181 (40.95)
Diabetes with complications	89 (5.58)	43 (9.73)
Chronic kidney disease	253 (15.86)	104 (23.53)
Malignancy	97 (6.08)	39 (8.82)
Metastatic cancer	17 (1.07)	9 (2.04)
Tobacco use	609 (41.07)	201 (50.63)
Substance use	146 (10.27)	40 (10.2)
Neighborhood characteristics, % (SD)		
Bachelor’s degree or higher	19 (14)	16 (12)
Less than high school	9 (6)	10 (6)
Median income (Q1–Q3)	48,680 (30,050–72,434)	40,488 (27,679–62,992)
Renters	37 (22)	39 (21)
Unemployment	5 (3)	5 (4)
Poverty rate	23 (18)	26 (18)
SNAP recipients	23 (18)	26 (17)
No vehicle access	14 (13)	16 (13)

*Q1–Q3*, first quartile–third quartile; *SD*, standard deviation; *IMV*, invasive mechanical ventilation; *BMI*, body mass index; *COPD*, chronic obstructive pulmonary disease; *SNAP*, Supplemental Nutrition Assistance Program

**Table 4:** Socioeconomic and Environmental Characteristics of Patients Requiring ICU Admission

Characteristic	No ICU (*n* = 1452)	ICU (*n* = 586)
Age, mean (SD), years	63.5 (17.0)	65.1 (14.2)
Female, no. (%)	767 (52.86)	260 (44.37)
Length of stay, days (Q1–Q3)	5 (3–8)	17 (23–62)
Readmission, no. (%)	160 (11.02)	66 (11.26)
Race, no. (%)		
White	520 (35.81)	174 (29.69)
Black	829 (57.09)	380 (64.85)
Asian/Pacific Islander	26 (1.79)	13 (2.22)
Native American	2 (0.14)	0 (0)
Other/missing	75 (5.17)	19 (3.24)
Living situation, no. (%)		
With family	801 (63.93)	409 (71.25)
Alone	190 (15.16)	77 (13.41)
Group facility	262 (20.91)	88 (15.33)
Insurance status, no. (%)		
Commercial insurance	392 (30.2)	102 (18.99)
Medicare	633 (48.77)	325 (60.52)
Medicaid	213 (16.41)	91 (16.95)
Self-pay	4 (0.31)	0 (0)
Other	17 (1.31)	4 (0.74)
No insurance	39 (3)	15 (2.79)
Medical conditions, no. (%)		
BMI, mean (SD), kg/m^2^	31.2 (8.2)	33.1 (9.5)
Charlson Comorbidity Index, mean (SD)	1.99 (2.05)	2.80 (2.39)
Hypertension	1019 (70.23)	475 (81.06)
Psychiatric condition	365 (25.16)	155 (26.45)
Coronary artery disease	191 (13.16)	117 (19.97)
Congestive heart failure	173 (11.92)	103 (17.58)
Cerebrovascular disease	188 (12.96)	83 (14.16)
COPD	169 (11.65)	125 (21.33)
Diabetes	423 (29.15)	229 (39.08)
Diabetes with complications	74 (5.1)	58 (9.9)
Chronic kidney disease	220 (15.16)	137 (23.38)
Malignancy	88 (6.06)	48 (8.19)
Metastatic cancer	14 (0.96)	12 (2.05)
Tobacco use	549 (40.7)	261 (49.15)
Substance use	134 (10.35)	52 (10.02)
Neighborhood characteristics, % (SD)		
Bachelor’s degree or higher	19 (14)	15 (12)
Less than high school	9 (6)	10 (6)
Median income (Q1–Q3)	49,867 (30,417–72,868)	38,060 (26,602–61,111)
Renters	36 (22)	40 (21)
Unemployment	4 (3)	5 (4)
Poverty rate	22 (18)	27 (18)
SNAP recipients	23 (17)	27 (17)
No vehicle access	14 (13)	17 (13)

*Q1–Q3*, first quartile–third quartile; *SD*, standard deviation; *ICU*, intensive care unit; *BMI*, body mass index; *COPD*, chronic obstructive pulmonary disease; *SNAP*, Supplemental Nutrition Assistance Program

### Neighborhood Effects

Patients who died did not live in significantly disadvantaged neighborhoods, with regard to median income, unemployment, and educational attainment (Table 1). However, patients who received IMV and ICU care lived in neighborhoods with significantly lower median income, educational attainment, and vehicle access, and higher unemployment rates and food insecurity (Tables 2 and 3). Black patients lived in significantly poorer neighborhoods than White patients (median income: $34,758 (24,531–56,095) vs. $63,317 (49,850–85,776), *p* < 0.001) (Table 4). The neighborhoods that Black patients lived in also reported lower educational attainment (bachelor’s degree or higher: 15.2% vs. 21.9%, *p* < 0.001), higher percentage of renters (43.9% vs. 25.9%, *p* < 0.001), higher unemployment rate (5.8% vs. 2.8%, *p* < 0.001), higher poverty rate (29.2% vs. 12.6%, *p* < 0.001), more Supplemental Nutrition Assistance Program (SNAP) benefits recipients (30.4% vs. 13.1%, *p* < 0.001), and more households without vehicle access (18.6% vs. 7.4%, *p* < 0.001). Black patients were almost twice as likely as White patients to have Medicaid insurance (19.4% vs. 11.2%, *p* < 0.001).

Analysis of outcomes by median income quartiles showed that patients from poorer neighborhoods had significantly higher frequencies of mechanical ventilation and ICU admission compared to patients from wealthier neighborhoods (Table 5). Stratification by Black or White race and median income quartiles revealed that among Black patients, those who lived in poorer neighborhoods had higher frequencies of death, mechanical ventilation, and ICU admission compared to Black patients living in wealthier neighborhoods. Among White patients, those living in disadvantaged neighborhoods had higher rates of ICU admission but similar rates of death and mechanical ventilation, when compared to White patients living in wealthier neighborhoods.

**Table 5:** Clinical Outcomes Stratified by Neighborhood Median Income Quartiles

	≤ $29.5K	$29.6K–47.6K	$47.6K–$68.9K	≥ $68.9K	*p* value
All patients					
Death, no. (%)	110 (21.7%)	104 (20.6%)	123 (24.1%)	105 (20.7%)	0.489
IMV, no. (%)	130 (25.4%)	126 (24.8%)	105 (20.5%)	81 (16.0%)	< 0.001
ICU, no. (%)	180 (35.2%)	170 (33.5%)	135 (26.4%)	101 (19.9%)	< 0.001
White only					
Death, no. (%)	13 (34.2%)	26 (25.2%)	88 (32.6%)	74 (26.3%)	0.276
IMV, no. (%)	11 (29.0%)	24 (23.1%)	54 (19.9%)	48 (17.1%)	0.259
ICU, no. (%)	16 (42.1%)	29 (27.9%)	68 (25.1%)	61 (21.7%)	0.046
Black only					
Death, no. (%)	92 (20.8%)	74 (20.4%)	30 (13.8%)	22 (12.3%)	0.017
IMV, no. (%)	116 (25.9%)	96 (26.4%)	48 (22.0%)	25 (14.0%)	0.006
ICU, no. (%)	159 (35.5%)	132 (36.3%)	61 (28.0%)	28 (15.6%)	< 0.001

*IMV*, invasive mechanical ventilation; *ICU*, intensive care unit

### Multivariate Analysis of Risk Factors for Mortality and Severe Disease

Table 6 shows the results of multivariate analyses adjusting for baseline characteristics. We used a model incorporating age (1-year increase), male sex, Black race, CCI (1-unit increase), obesity (BMI > 35 kg/m^2^), smoking status, neighborhood median income ($10,000 increase), and living in a group facility. Increased age, male sex, higher CCI, and living in a group facility were predictors of death. Black race was not a significant predictor of death, even though Black patients had a lower rate of death than White patients.

**Table 6:** Multivariable Analysis of Characteristics Associated with Death, Mechanical Ventilation, and ICU Admission

**Characteristic**	**Death**		**Mechanical ventilation**		**ICU admission**	
	**Adjusted OR (95% CI)**	***p*** value	**Adjusted OR (95% CI)**	***p*** value	**Adjusted OR (95% CI)**	***p*** value
Age (1-year increase)	1.06 (1.04, 1.07)	< 0.001	1.01 (0.99, 1.02)	0.162	1.00 (0.99, 1.01)	0.589
Male sex	1.47 (1.14, 1.90)	0.003	1.63 (1.29, 2.07)	< 0.001	1.55 (1.25, 1.92)	< 0.001
Black race	0.86 (0.64, 1.14)	0.284	1.16 (0.89, 1.51)	0.284	1.13 (0.88, 1.44)	0.342
CCI (1-unit increase)	1.23 (1.16, 1.30)	< 0.001	1.13 (1.07, 1.19)	< 0.001	1.16 (1.10, 1.22)	< 0.001
BMI > 35	0.96 (0.69, 1.32)	0.78	1.64 (1.26, 2.14)	< 0.001	1.51 (1.19, 1.94)	< 0.001
Income ($10,000 increase)	0.97 (0.92, 1.01)	0.14	0.95 (0.91, 0.99)	0.02	0.92 (0.89, 0.96)	< 0.001
Tobacco use	1.08 (0.84, 1.40)	0.547	1.20 (0.94, 1.53)	0.145	1.13 (0.91, 1.41)	0.279
Group facility	1.61 (1.19, 2.16)	0.002	0.63 (0.45, 0.88)	0.007	0.64 (0.47, 0.87)	0.004
**White patients**	**Death**		**Mechanical ventilation**		**ICU admission**	
**Variable**	**Adjusted OR (95% CI)**	***p*** value	**Adjusted OR (95% CI)**	***p*** value	**Adjusted OR (95% CI)**	***p*** value
Age (1-year increase)	1.04 (1.03, 1.06)	< 0.001	1.00 (0.99, 1.02)	0.858	0.99 (0.98, 1.01)	0.541
Male sex	1.24 (0.83, 1.85)	0.298	1.47 (0.97, 2.22)	0.068	1.39 (0.95, 2.02)	0.092
CCI (1-unit increase)	1.22 (1.11, 1.34)	< 0.001	1.13 (1.03, 1.25)	0.012	1.14 (1.04, 1.25)	0.004
BMI > 35	0.44 (0.23, 0.82)	0.01	1.42 (0.86, 2.340	0.175	1.26 (0.79, 2.00)	0.339
Income ($10,000 increase)	0.97 (0.90, 1.04)	0.344	0.94 (0.87, 1.02)	0.131	0.93 (0.86, 0.99)	0.033
Tobacco use	0.84 (0.56, 1.25)	0.382	1.16 (0.76, 1.75)	0.5	1.35 (0.92, 1.97)	0.13
Lives in group facility	1.92 (1.26, 2.94)	0.003	0.72 (0.44, 1.17)	0.184	0.74 (0.47, 1.16)	0.193
**Black patients**	**Death**		**Mechanical ventilation**		**ICU admission**	
**Variable**	**Adjusted OR (95% CI)**	***p*** value	**Adjusted OR (95% CI)**	***p*** value	**Adjusted OR (95% CI)**	***p*** value
Age (1-year increase)	1.06 (1.04, 1.07)	< 0.001	1.01 (0.99, 1.02)	0.196	1.00 (0.99, 1.02)	0.421
Male sex	1.76 (1.24, 2.51)	0.002	1.76 (1.30, 2.37)	< 0.001	1.66 (1.26, 2.19)	< 0.001
CCI (1-unit increase)	1.28 (1.18, 1.38)	< 0.001	1.14 (1.07, 1.22)	< 0.001	1.18 (1.10, 1.26)	< 0.001
BMI > 35	1.35 (0.90, 2.02)	0.15	1.74 (1.26, 2.41)	< 0.001	1.62 (1.20, 2.20)	0.002
Income ($10,000 increase)	0.93 (0.87, 0.99)	0.032	0.93 (0.88, 0.99)	0.023	0.89 (0.85, 0.95)	< 0.001
Tobacco use	1.33 (0.93, 1.90)	0.118	1.26 (0.92, 1.72)	0.149	1.08 (0.81, 1.44)	0.603
Lives in group facility	1.23 (0.78, 1.94)	0.38	0.59 (0.36, 0.95)	0.031	0.61 (0.39, 0.93)	0.023

*CCI*, Charlson Comorbidity Index; *BMI*, body mass index; *Income*, neighborhood median income

Male sex, increasing CCI, obesity, and lower neighborhood median income were significant predictors for IMV and ICU admission, while living in a group facility was protective against IMV and ICU admission. Age was not an independent predictor of IMV or ICU. Black race was not an independent predictor of IMV or ICU, even though Black patients had higher rates of mechanical ventilation and ICU admission.

To determine race-specific predictors, we stratified our model by Black and White race (Table 6). Interestingly, male sex was an independent predictor for death, IMV, and ICU admission in Black patients, but not in White patients. Neighborhood median income was a significant predictor for death, IMV and ICU admission in Black patients, but only ICU admission in White patients. Living in a group facility was a predictor for death in White patients, but not Black patients.

## DISCUSSION

The COVID-19 pandemic revealed the health disparities that exist between the Black and White populations of southeast Michigan, a population which can be generalized to other metropolitan areas. This study aims to shed light on the characteristics of these disparities by correlating outcomes with race and socioeconomic factors. An earlier case series conducted by our colleagues described the clinical characteristics and outcomes for the first wave of COVID-19 patients in metropolitan Detroit.^8^ Our study builds on their work while factoring in the socioeconomic variables of a large cohort hospitalized with COVID-19 during the peak of the pandemic in Michigan. Consistent with our results, they conclude that most of the infected hospitalized patients from the first wave of the pandemic were Black. In our study, during the peak of the pandemic, Black patients comprised nearly 60% of our cohort, despite making up only 14% of the state’s population. This overrepresentation is supported by Black patients representing a larger proportion of each hospital’s admissions than respective city demographics. In our study, the CFR was higher in White patients than in Black patients (29.1% vs. 18.1%), which is likely due to the significantly older age of White patients compared to Black patients (68.6 years vs. 61.7 years). The lower CFR for Black patients suggests that the higher death rate of COVID-19 among the Black population per capita in Michigan and nationally may stem from higher rates of infection per capita, which is supported by analyses of infection rates by race.^21,22^

Social distancing and self-isolation are highly effective in reducing transmission but remain a difficulty for many Black Americans due to housing density, frontline employment, and food deserts, all of which are linked to socioeconomic disparities.^23–25^ Frontline employment comprised a plurality of patients under 65 years, though Black and White patients in our cohort had similar employment characteristics. Finally, we found that living in a group facility is an independent predictor for mortality, even after adjusting for age and comorbidities. This is again, likely due to sharing space and little to no opportunity for proper social distancing and isolation, highlighting the need for reform. Our findings highlight the importance of the living and social environment on COVID-19 outcomes and is reflected in the devastation that the pandemic has wrought upon nursing facilities in southeast Michigan and impoverished communities.^26,27^

Multivariable analysis found increased age, male sex, and comorbidities but not race to be significant predictors of mortality, consistent with other reports.^5,7–10^ Hypertension, cardiovascular disease, COPD, diabetes, CKD, and cancer were associated with death, as was increased CCI. Although CCI for White and Black patients were similar, the comorbidities disproportionately prevalent in the Black population—hypertension, diabetes, and CKD—were associated with a worse prognosis. The higher prevalence of these conditions did not result in higher mortality in hospitalized Black patients, but may account for higher hospital admission rates, consistent with comparative analyses of hospitalized and discharged patients.^7,8^ In the multivariate analysis, Black race was not a significant predictor for IMV or ICU admission, consistent with analyses from an earlier study in our health system.^8^ Obesity was an independent predictor of need for IMV and ICU care, consistent with other reports.^28,29^ Notably, Black patients had significantly higher BMI than White patients (BMI: 33.0 vs. 29.8, *p* < 0.001). Higher rates of obesity among the Black population in the USA have been linked to higher prevalence of food deserts and food insecurity in communities of color.^30–32^ Finally, the finding that male sex was a more significant predictor in Black patients highlights the unique stresses of the Black male experience in the USA, many of which were not captured in our analysis. These stresses include police violence, mass incarceration, and health and education disparities.^33–38^

When neighborhood income quartiles were stratified by race, poorer Black patients were at higher risk of death, mechanical ventilation, and ICU admission. This study is the first to identify median income as an independent predictor of IMV and ICU admission in COVID-19, even after adjusting for age, sex, and comorbidities. Analysis stratified by race revealed that male sex, obesity, and lower neighborhood median income were more significant predictors of poor outcomes for Black patients than for White patients. The disproportionate impact of neighborhood disadvantage on Black patients is likely due to the large income disparity between Black and White patients (neighborhood median income: $34,758 vs. $63,317, *p* < 0.001). We ground these income disparities on the structural inequities that drive the racial income and wealth gap in the USA.^39–41^

Our findings indicate that the racial disparities associated with the COVID-19 pandemic are not due to intrinsic characteristics of racial groups, but rather are produced by systemic inequities rooted in structural racism. These inequities affect income, educational attainment, food security, and healthcare. Therefore, social and legislative policies are critical in order to reduce socioeconomic disparities experienced by Black, Indigenous, and people of color (BIPOC) and can inform emerging initiatives such as the recently formed Michigan Coronavirus Task Force on Racial Disparities and Black Leadership Advisory Council.^42,43^ Solutions should be multi-level, synchronous, and coordinated. An intervention consisting of focused testing efforts with culturally competent strategies and messaging should target the most disadvantaged areas, which can be identified through the census. Upon hospital admission, appropriate screening for SDOH should be conducted to inform discharge planning, whereby patients from disadvantaged neighborhoods are provided additional resources for support. The healthcare system should also strive to better understand and eliminate institutional and clinician biases and address systemic racism in healthcare that hinders disease management and preventive care in underserved communities.^44–46^

Our study has limitations worth noting. Primarily, the study was retrospective in nature and collected data from one integrated healthcare system in southeast Michigan, limiting generalizability to other locations. Although this study examines the impact of race in COVID-19 outcomes, it focuses mainly on Black and White patients. Hispanic patients and non-English speakers made up a small percentage of the cohort, limiting the conclusions that could be drawn for those subpopulations. There are large Middle Eastern, Latinx, and South Asian communities in metropolitan Detroit that were also not explicitly accounted for in the study. Further studies or larger cohorts could provide additional insight into the role of not only race but also ethnicity on COVID-19 outcomes. Another limitation is our use of census tract median income as a surrogate measure of neighborhood disadvantage. Though median income correlates with other socioeconomic measures on the census tract level, there may be high variability within a census tract that we did not account for. We were unable to obtain socioeconomic measures on the individual level and census tract socioeconomic measures may not accurately portray each individual’s situation, which may change the variance in our effect estimates.

Our study examines one of the largest cohorts of hospitalized COVID-19 patients in an urban area with in-depth focus on the systemic inequities that impact social determinants of health. Detroit is home to one of the largest populations of Black Americans in the USA, and some of the nation’s largest racial and health disparities, making it the ideal location to study and identify solutions to these deep-seated problems. The collection of social and environmental characteristics in our study sheds light on the large socioeconomic differences between Black and White patients with COVID-19. We identify neighborhood median income as a significant and independent predictor of severe disease, highlighting the need for multi-level approaches to reduce these disparities.

## Supplementary Information

ESM 1 (DOCX 34 kb)
