# Supplementary material for: Impact of Race and Socioeconomic Status on Outcomes in Patients Hospitalized with COVID-19
Source: J Gen Intern Med. 2021 Jan 27;36(5):1302–9. doi: 10.1007/s11606-020-06527-1 (PMC7840076; doi:10.1007/s11606-020-06527-1)
Supplement: Supplementary file 1 — (DOCX 34 kb) [file 11606_2020_6527_MOESM1_ESM.docx]

**Appendix Table 1. Definition of Covariates.**

| **Variable** | **Type of variable** | **Categories** |
| --- | --- | --- |
| Age | Continuous |  |
| Female | Dichotomous |  |
| Length of stay | Continuous |  |
| Readmission | Dichotomous |  |
| Race | Categorical | White, Black, Asian/Pacific Islander, Native American, Other/missing |
| Living situation | Categorical | With family, Alone, Group facility |
| Insurance status | Categorical | Commercial insurance, Medicare, Medicaid, Self-pay, Other, No insurance |
| BMI | Continuous |  |
| Charlson Comorbidity Index | Continuous |  |
| Hypertension | Dichotomous |  |
| Psychiatric condition | Dichotomous |  |
| Coronary artery disease | Dichotomous |  |
| Congestive heart failure | Dichotomous |  |
| Cerebrovascular disease | Dichotomous |  |
| COPD | Dichotomous |  |
| Diabetes | Dichotomous |  |
| Diabetes with complications | Dichotomous |  |
| Chronic kidney disease | Dichotomous |  |
| Malignancy | Dichotomous |  |
| Metastatic cancer | Dichotomous |  |
| Tobacco use | Dichotomous |  |
| Substance use | Dichotomous |  |
| Bachelor's degree or higher | Continuous |  |
| Less than high school | Continuous |  |
| Median income | Continuous |  |
| Renters | Continuous |  |
| Unemployment | Continuous |  |
| Poverty rate | Continuous |  |
| SNAP recipients | Continuous |  |
| No vehicle access | Continuous |  |

**Appendix Table 2. Presenting Characteristics and Hospital Course for Alive and Dead Patients.**

| **Characteristic** | **All (n=2038)** | **Alive (n=1587)** | **Dead (n=442)** |
| --- | --- | --- | --- |
| **Symptoms, no. (%)** |  |  |  |
| Duration of symptoms, days (Q1-Q3) | 6 (3-8) | 7 (3-9) | 6 (2-7) |
| Altered mental status | 320 (16.8) | 166 (11.05) | 153 (39.13) |
| Fever | 1086 (60.4) | 909 (62.05) | 172 (53.25) |
| Chills | 589 (39.0) | 519 (40.77) | 68 (29.82) |
| Cough | 1372 (79.1) | 1142 (80.08) | 226 (75.08) |
| Dyspnea | 1455 (78.4) | 1133 (76.81) | 315 (84.68) |
| Headache | 165 (12.0) | 155 (13.32) | 10 (4.76) |
| Fatigue | 836 (71.3) | 690 (70.19) | 140 (76.5) |
| Myalgia | 458 (37.9) | 410 (40.63) | 46 (23.71) |
| Nausea/vomiting/diarrhea | 661 (39.5) | 572 (40.97) | 87 (32.34) |
| **Vital signs, mean (SD)** |  |  |  |
| Temperature, Celsius | 37.4 (2.8) | 37.4 (2.7) | 37.4 (3.2) |
| Heart rate, BPM | 94 (19) | 95 (18) | 93 (21) |
| Systolic blood pressure, mmHg | 131 (24) | 131 (23) | 128 (27) |
| Diastolic blood pressure, mmHg | 74 (15) | 75 (15) | 71 (16) |
| Respiratory rate, BPM | 22 (6) | 21 (6) | 23 (7) |
| SpO2, % | 95 (5) | 95 (4) | 94 (7) |
| Received supplemental O2 in ED, no. (%) | 1312 (64.7) | 924 (58.48) | 380 (86.76) |
| qSOFA | 0.65 (0.69) | 0.54 (0.62) | 1.02 (0.81) |
| **Laboratory values, median (Q1-Q3)** |  |  |  |
| White blood cell, cells/uL | 6.5 (4.8-9.3) | 6.3 (4.7-8.7) | 7.6 (5.4-11.2) |
| Absolute neutrophil count (cells/uL) | 5.0 (3.4-7.4) | 4.7 (3.3-6.9) | 6.1 (4.1-9.6) |
| Absolute lymphocyte count (cells/uL) | 0.8 (0.6-1.2) | 0.9 (0.6-1.2) | 0.7 (0.5-1.0) |
| Procalcitonin, ng/mL | 0.16 (0.10-0.47) | 0.13 (0.10-0.32) | 0.40 (0.16-1.32) |
| D-dimer, ug/mL | 1.30 (0.71-2.58) | 1.12 (0.64-2.24) | 2.19 (1.19-3.95) |
| Brain natriuretic peptide, pg/mL | 48 (22-140) | 37 (19-90) | 134 (55-371) |
| Ferritin, ng/mL | 500 (238-993) | 458 (221-923) | 708 (328-1316) |
| C-reactive protein, mg/L | 9.3 (4.5-15.1) | 8.5 (3.7-13.9) | 11.9 (7.0-18.6) |
| Lactate dehydrogenase, IU/L | 318 (238-429) | 306 (234-412) | 363 (259-512) |
| Elevated troponin, no. (%) | 784 (39.7) | 469 (30.45) | 310 (72.43) |
| Aspartate aminotransferase, IU/L | 40 (28-62) | 38 (27-59) | 48 (30-76) |
| Alanine aminotransferase, IU/L | 26 (17-42) | 26 (18-43) | 25 (17-40) |
| Creatinine, mg/dL | 1.14 (0.85-1.71) | 1.06 (0.82-1.54) | 1.52 (1.06-2.54) |
| **Hospital course and treatment, no. (%)** |  |  |  |
| Acute kidney injury | 823 (40.5) | 519 (32.74) | 296 (67.58) |
| Acute hepatic injury | 62 (3.1) | 26 (1.67) | 36 (8.35) |
| Renal replacement therapy | 194 (9.5) | 105 (6.62) | 84 (19) |
| Hydroxychloroquine | 1645 (80.7) | 1310 (82.55) | 327 (73.98) |
| Systemic steroids | 1505 (73.8) | 1141 (71.9) | 355 (80.32) |
| Antibiotics | 1668 (81.8) | 1254 (79.02) | 405 (91.63) |
| Remdesivir | 28 (1.4) | 17 (1.07) | 10 (2.26) |
| Tocilizumab | 118 (5.8) | 76 (4.79) | 42 (9.5) |
| Treatment dose anticoagulation | 477 (23.4) | 280 (17.64) | 190 (42.99) |

**Appendix Table 3. Presenting Characteristics and Hospital Course for Patients Requiring Mechanical Ventilation.**

| **Characteristic** | **No IMV (n = 1596)** | **IMV (n = 442)** |
| --- | --- | --- |
| **Symptoms, no. (%)** |  |  |
| Duration of symptoms, days (Q1-Q3) | 7 (3-9) | 5 (3-7) |
| Altered mental status | 226 (15.1) | 94 (23.21) |
| Fever | 842 (58.76) | 244 (67.03) |
| Chills | 469 (38.47) | 120 (41.38) |
| Cough | 1087 (78.54) | 285 (81.43) |
| Dyspnea | 1086 (75.1) | 369 (90.22) |
| Headache | 139 (12.46) | 26 (9.85) |
| Fatigue | 678 (70.55) | 158 (74.88) |
| Myalgia | 375 (38.66) | 83 (34.73) |
| Nausea/vomiting/diarrhea | 537 (39.78) | 124 (38.39) |
| **Vital signs, mean (SD)** |  |  |
| Temperature, Celsius | 37.4 (2.7) | 37.6 (3.2) |
| Heart rate, BPM | 94 (19) | 96 (19) |
| Systolic blood pressure, mmHg | 131 (24) | 130 (26) |
| Diastolic blood pressure, mmHg | 74 (15) | 73 (15) |
| Respiratory rate, BPM | 21 (6) | 24 (7) |
| SpO2, % | 95 (4) | 94 (7) |
| Received supplemental O2 in ED, no. (%) | 915 (57.62) | 397 (90.43) |
| qSOFA | 0.57 (0.67) | 0.92 (0.72) |
| **Laboratory values, median (Q1-Q3)** | |  |
| White blood cell, cells/uL | 6.3 (4.7-9.0) | 7.5 (5.4-10.5) |
| Absolute neutrophil count (cells/uL) | 4.7 (3.3-7.1) | 5.9 (4.1-8.6) |
| Absolute lymphocyte count (cells/uL) | 0.9 (0.6-1.2) | 0.8 (0.5-1.1) |
| Procalcitonin, ng/mL | 0.13 (0.10-0.32) | 0.39 (0.15-1.29) |
| D-dimer, ug/mL | 1.19 (0.65-2.30) | 1.83 (0.99-3.78) |
| Brain natriuretic peptide, pg/mL | 42 (20-117) | 80 (33-242) |
| Ferritin, ng/mL | 450 (221-895) | 773 (323-1384) |
| C-reactive protein, mg/L | 8.2 (3.6-13.7) | 12.3 (7.7-19.1) |
| Lactate dehydrogenase, IU/L | 298 (227-394) | 413 (302-577) |
| Elevated troponin, no. (%) | 514 (33.44) | 270 (61.36) |
| Aspartate aminotransferase, IU/L | 37 (27-57) | 50 (34-81) |
| Alanine aminotransferase, IU/L | 25 (17-40) | 30 (19-47) |
| Creatinine, mg/dL | 1.08 (0.83-1.60) | 1.39 (1.00-2.19) |
| **Hospital course and treatment, no. (%)** | |  |
| Acute kidney injury | 497 (31.26) | 326 (73.76) |
| Acute hepatic injury | 15 (0.97) | 47 (10.63) |
| Renal replacement therapy | 78 (4.89) | 116 (26.24) |
| Hydroxychloroquine | 1242 (77.82) | 403 (91.18) |
| Systemic steroids | 1090 (68.3) | 415 (93.89) |
| Antibiotics | 1237 (77.51) | 431 (97.51) |
| Remdesivir | 5 (0.31) | 23 (5.2) |
| Tocilizumab | 23 (1.44) | 95 (21.49) |
| Treatment dose anticoagulation | 226 (14.16) | 251 (56.79) |

**Appendix Table 4. Presenting Characteristics and Hospital Course for Patients Requiring ICU Admission.**

| **Characteristic** | **No ICU (n = 1452)** | **ICU (n = 586)** |
| --- | --- | --- |
| **Symptoms, no. (%)** |  |  |
| Duration of symptoms, days (Q1-Q3) | 7 (3-9) | 5 (3-7) |
| Altered mental status | 200 (14.68) | 120 (22.22) |
| Fever | 778 (59.53) | 308 (62.86) |
| Chills | 435 (38.98) | 154 (39.19) |
| Cough | 989 (78.62) | 383 (80.46) |
| Dyspnea | 990 (75) | 465 (86.92) |
| Headache | 128 (12.51) | 37 (10.36) |
| Fatigue | 626 (71.38) | 210 (71.19) |
| Myalgia | 342 (39) | 116 (34.94) |
| Nausea/vomiting/diarrhea | 490 (39.81) | 171 (38.69) |
| **Vital signs, mean (SD)** |  |  |
| Temperature, Celsius | 37.4 (2.8) | 37.5 (2.8) |
| Heart rate, BPM | 94 (19) | 96 (19) |
| Systolic blood pressure, mmHg | 131 (24) | 129 (26) |
| Diastolic blood pressure, mmHg | 74 (15) | 73 (16) |
| Respiratory rate, BPM | 21 (5) | 24 (7) |
| SpO2, % | 95 (4) | 94 (7) |
| Received supplemental O2 in ED, no. (%) | 801 (55.47) | 511 (87.65) |
| qSOFA | 0.56 (0.66) | 0.87 (0.72) |
| **Laboratory values, median (Q1-Q3)** |  |  |
| White blood cell, cells/uL | 6.2 (4.7-8.6) | 7.5 (5.4-10.8) |
| Absolute neutrophil count (cells/uL) | 4.6 (3.3-6.8) | 7.0 (4.1-8.9) |
| Absolute lymphocyte count (cells/uL) | 0.9 (0.6-1.2) | 0.8 (0.5-1.1) |
| Procalcitonin, ng/mL | 0.13 (0.10-0.30) | 0.35 (0.14-1.08) |
| D-dimer, ug/mL | 1.12 (0.64-2.24) | 1.79 (0.98-3.69) |
| Brain natriuretic peptide, pg/mL | 40 (19-106) | 76 (31-215) |
| Ferritin, ng/mL | 443 (218-882) | 718 (318-1338) |
| C-reactive protein, mg/L | 7.9 (3.3-13.3) | 11.9 (7.2-18.8) |
| Lactate dehydrogenase, IU/L | 290 (224-385) | 394 (295-548) |
| Elevated troponin, no. (%) | 451 (32.31) | 333 (57.31) |
| Aspartate aminotransferase, IU/L | 37 (27-57) | 47 (31-77) |
| Alanine aminotransferase, IU/L | 25 (17-40) | 28 (18-46) |
| Creatinine, mg/dL | 1.06 (0.82-1.55) | 1.38 (0.99-2.19) |
| **Hospital course and treatment, no. (%)** | |  |
| Acute kidney injury | 428 (29.6) | 395 (67.41) |
| Acute hepatic injury | 12 (0.85) | 50 (8.56) |
| Renal replacement therapy | 65 (4.48) | 129 (22.01) |
| Hydroxychloroquine | 1126 (77.55) | 519 (88.57) |
| Systemic steroids | 966 (66.53) | 539 (91.98) |
| Antibiotics | 1104 (76.03) | 564 (96.25) |
| Remdesivir | 4 (0.28) | 24 (4.1) |
| Tocilizumab | 19 (1.31) | 99 (16.89) |
| Treatment dose anticoagulation | 186 (12.81) | 291 (49.66) |

**Appendix Table 5. Presenting Characteristics and Hospital Course for Black and White Patients.**

| **Characteristic** | **White (n = 694)** | **Black (n = 1209)** |
| --- | --- | --- |
| **Symptoms, no. (%)** |  |  |
| Duration of symptoms, days (Q1-Q3) | 6 (3-9) | 6 (3-8) |
| Altered mental status | 134 (21.51) | 168 (14.62) |
| Fever | 355 (61.85) | 646 (59.05) |
| Chills | 181 (37.95) | 372 (40.35) |
| Cough | 444 (81.02) | 823 (77.5) |
| Dyspnea | 479 (77.63) | 874 (78.67) |
| Headache | 37 (8.71) | 115 (13.39) |
| Fatigue | 281 (75.13) | 493 (68.66) |
| Myalgia | 139 (38.5) | 287 (37.66) |
| Nausea/vomiting/diarrhea | 182 (35.14) | 424 (41.01) |
| **Vital signs, mean (SD)** |  |  |
| Temperature, Celsius | 37.3 (2.6) | 37.5 (3.0) |
| Heart rate, BPM | 91 (19) | 96 (19) |
| Systolic blood pressure, mmHg | 131 (24) | 130 (24) |
| Diastolic blood pressure, mmHg | 72 (16) | 75 (15) |
| Respiratory rate, BPM | 21 (6) | 22 (6) |
| SpO2, % | 95 (4) | 95 (6) |
| Received supplemental O2 in ED, no. (%) | 453 (65.56) | 772 (64.23) |
| qSOFA | 0.65 (0.71) | 0.65 (0.69) |
| **Laboratory values, median (Q1-Q3)** | |  |
| White blood cell, cells/uL | 6.6 (4.8-9.6) | 6.5 (4.8-9.1) |
| Absolute neutrophil count (cells/uL) | 5.0 (3.5-7.7) | 4.9 (3.4-7.4) |
| Absolute lymphocyte count (cells/uL) | 0.8 (0.5-1.2) | 0.9 (0.6-1.2) |
| Procalcitonin, ng/mL | 0.14 (0.10-0.34) | 0.19 (0.10-0.57) |
| D-dimer, ug/mL | 1.27 (0.71-2.52) | 1.39 (0.74-2.78) |
| Brain natriuretic peptide, pg/mL | 79 (30-203) | 39 (19-106) |
| Ferritin, ng/mL | 419 (215-837) | 537 (263-1119) |
| C-reactive protein, mg/L | 8.9 (4.1-13.9) | 9.5 (4.7-15.7) |
| Lactate dehydrogenase, IU/L | 265 (204-349) | 350 (261-481) |
| Elevated troponin, no. (%) | 257 (38.76) | 491 (41.61) |
| Aspartate aminotransferase, IU/L | 39 (27-59) | 41 (28-63) |
| Alanine aminotransferase, IU/L | 27 (19-44) | 25 (16-40) |
| Creatinine, mg/dL | 1.04 (0.80-1.51) | 1.21 (0.90-1.86) |
| **Hospital course, no. (%)** |  |  |
| Acute kidney injury | 241 (34.98) | 536 (44.37) |
| Acute hepatic injury | 19 (2.83) | 38 (3.2) |
| Renal replacement therapy | 38 (5.48) | 148 (12.25) |
| Hydroxychloroquine | 525 (75.65) | 1001 (82.8) |
| Systemic steroids | 490 (70.61) | 915 (75.68) |
| Antibiotics | 569 (81.99) | 995 (82.3) |
| Remdesivir | 5 (0.72) | 21 (1.74) |
| Tocilizumab | 47 (6.77) | 62 (5.13) |
| Treatment dose anticoagulation | 184 (26.51) | 273 (22.58) |
